# Factores asociados al cumplimiento de la terapia preventiva con isoniacida en niños en Quito, Ecuador (2014-2016 y 2018)

**DOI:** 10.26633/RPSP.2019.97

**Published:** 2019-12-20

**Authors:** Adriana Chacón, Edith Alarcón, Lucelly López

**Affiliations:** 1 Ministerio de Salud Pública Distrito de Salud 17D08 Quito Ecuador Ministerio de Salud Pública, Distrito de Salud 17D08, Quito, Ecuador.; 2 Organización Panamericana de la Salud Organización Panamericana de la Salud Washington D.C. Estados Unidos de América Organización Panamericana de la Salud, Washington D.C., Estados Unidos de América.; 3 Universidad Pontificia Bolivariana Universidad Pontificia Bolivariana Medellín Colombia Universidad Pontificia Bolivariana, Medellín, Colombia.

**Keywords:** Tuberculosis, terapia preventiva, isoniacida, investigación operativa, Ecuador, Tuberculosis, prevention & control, isoniazid, operations research, Ecuador, Tuberculose, prevenção & controle, isoniazida, pesquisa operacional, Ecuador

## Abstract

**Objetivo.:**

Conocer el porcentaje de cumplimiento de la terapia preventiva con isoniacida (TPI) en los establecimientos de salud de Quito, Ecuador y sus factores asociados en los niños menores de 5 años.

**Métodos.:**

Investigación operativa con diseño de cohorte, en la que se obtuvo datos de informes y tarjetas de administración de tratamiento de los niños en TPI de los años 2014 al 2016 y de encuestas *ad hoc* aplicadas a cuidadores de los niños que recibieron TPI durante el año 2018.

**Resultados.:**

Los niños menores de 5 años correspondieron a 29,3% del total de los contactos de los casos índices; 73% cumplieron TPI y 88,9% completaron al menos 6 meses de terapia. Se encontró asociación con la carga bacilar del caso índice, con la condición de pertenecer a un determinado distrito y su año de inicio. Se realizaron encuestas a 9 personas, funcionarios de los establecimientos salud y a 9 tutores de los niños; se registraron respuestas diversas sobre el agente causal de la tuberculosis, su transmisión y las características de la terapia preventiva.

**Conclusiones.:**

La mayoría de los niños menores de 5 años que iniciaron TPI cumplieron con al menos 80% de las dosis prescritas, con determinadas asociaciones y percepciones en los cuidadores. En este contexto, surge la necesidad de realizar nuevas investigaciones operativas, para indagar más ampliamente sobre la adherencia y sobre los conocimientos, actitudes y prácticas de los profesionales de salud, los afectados por tuberculosis y su entorno.

## Abstract (en)

**Objective.:**

Determine the percentage of children under 5 years of age who completed isoniazid preventive therapy (IPT) in health facilities in Quito, Ecuador, and assess related factors.

**Methods.:**

Operations research with cohort design. Data were obtained from treatment reports on children in IPT between 2014 and 2016, and from ad hoc surveys of caregivers of children who received IPT in 2018.

**Results.:**

Children under 5 represented 29.3% of all contacts of index cases; 73% completed IPT and 88.9% had at least six months of therapy. Associations were found with the bacterial load of the index case, with living in a given district, and with the year in which treatment was initiated. Surveys were conducted with nine staff members of health facilities and nine caregivers of children; diverse responses were given regarding the causative agent of tuberculosis, its transmission, and the characteristics of preventive therapy.

**Conclusions.:**

The majority of children under 5 years of age who initiated IPT completed at least 80% of the prescribed doses, with varying associations and knowledge on the part of their caregivers. In this context, there is a need for further operations research in order to learn more about adherence and about the knowledge, attitudes, and practices of health professionals and those affected by tuberculosis, and their environment.

## RESUMO

**Objetivo.:**

Conhecer a porcentagem de adesão à terapia preventiva com isoniazida (TPI) nas unidades de saúde de Quito, Equador, e os fatores associados à adesão em crianças com menos de 5 anos de idade.

**Métodos.:**

Pesquisa operacional com desenho de coorte, na qual foram obtidos dados de relatórios e dos cartões de administração de TPI em crianças entre 2014 e 2016, bem como de questionários *ad hoc* aplicados aos cuidadores das crianças que receberam TPI durante o ano de 2018.

**Resultados.:**

As crianças com menos de 5 anos de idade representaram 29,3% do total dos contatos dos casos índices; 73% aderiram à TPI e 88,9% completaram pelo menos 6 meses de tratamento. Identificamos associações com a carga bacilar do caso índice, com o distrito de residência do paciente e com o ano de início. Realizamos inquéritos com 9 funcionários das unidades de saúde e com 9 responsáveis pelas crianças, registrando respostas variadas sobre o agente causal da tuberculose, sua transmissão e as características da terapia preventiva.

**Conclusões.:**

Em sua maioria, as crianças com menos de 5 anos de idade que iniciaram a TPI aderiram a pelo menos 80% das doses prescritas, havendo associações com certos fatores e com os conhecimentos dos cuidadores. Neste contexto, fica clara a necessidade de realizar novos estudos operacionais para compreender melhor a adesão ao tratamento e os conhecimentos, atitudes e práticas dos profissionais da saúde, dos afetados pela tuberculose e das pessoas em seu entorno.

Según el Informe Mundial de Tuberculosis 2018, en el año 2017 se estimaron 10 millones de casos de tuberculosis (TB), con la notificación oficial de 6,4 millones, de los cuales 10% correspondieron a tuberculosis infantil (0 a 14 años). En la Región de las Américas, se estimaron un total de 282 000 casos de tuberculosis, de los cuales 33 000 son niños menores de 15 años (12%) (1). En el Ecuador, se estimaron 7 200 casos en total y 970 menores de 15 años (11,8%), con la notificación en el mismo período de 6 094 y 154 casos, lo que corresponde a 2,5% del total de casos nuevos y antes tratados (2), inferior a 7,1% reportado a nivel mundial. Estos datos muestran, en Ecuador, una menor transmisión a la población infantil o menor búsqueda y diagnóstico con relación al promedio regional; sin embargo, es necesario identificar la brecha de casos según la estimación de 10% del total, con base en la disponibilidad de recursos diagnósticos y asegurar estrategias que corten la cadena de transmisión, entre ellas la implementación y seguimiento de medidas preventivas.

En el mismo reporte publicado por la Organización Mundial de la Salud (OMS), se publicaron datos de 124 países sobre el inicio de terapia preventiva para tuberculosis en niños en el año 2017, con un total de 292 182 niños que iniciaron terapia preventiva. Esto muestra un incremento de 79% en relación con el año previo; sin embargo, este número corresponde solo a 23% de los niños estimados elegibles para esta terapia (1).

El estudio de contactos de los pacientes con tuberculosis se realiza con base en el riesgo de desarrollar esta enfermedad y las posibles consecuencias de la patología según la condición inmunológica de los expuestos al caso índice; en este sentido también se dirigen las acciones de intervención a los grupos prioritarios, con la inclusión de terapia preventiva con isoniacida (TPI) en niños menores de 5 años, en personas portadoras de virus de la inmunodeficiencia humana (VIH) y en otras patologías como aquellas que requieren el uso de biológicos (3).

La TPI está indicada en los niños menores de 5 años contactos de pacientes con tuberculosis (TB) pulmonar confirmada por bacteriología una vez se ha excluido enfermedad activa, debido a que en ellos aumenta el riesgo de progresión a enfermedad en 40% en menores de 12 meses y en 25% en los mayores de un año cuando existe mayor exposición con el enfermo con TB activa sin tratamiento (4).

Una revisión sistemática realizada entre 1996 y 2017 sobre el manejo de contactos menores de personas con TB identificó que se debe intervenir cada uno de los aspectos de la cascada a evaluar en el manejo de estos contactos, como la identificación, tamizaje e indicación del tratamiento preventivo y su cumplimiento, para disminuir las pérdidas en cada uno de estos pasos (5).

Existen factores que influyen en la adherencia a la terapia preventiva con isoniacida: aspectos socioeconómicos, relación con personas afectadas por tuberculosis (6), condiciones de manejo del país, facilidad de contar con formulaciones pediátricas y tener estructuradas estrategias educacionales, incluidos el apoyo y la supervisión por parte del personal de salud (7), migración, viajes (8) y la duración del tratamiento de infección latente de TB (9).

En estudios realizados sobre adherencia a la TPI por nueve meses, se ha determinado como cumplimiento que el niño haya recibido la TPI en al menos 80% del tiempo indicado de duración, también se han comprobado rangos más altos de adherencia en esquemas de tratamiento preventivo más corto (8, 9).

La TPI administrada con un cumplimiento adecuado debería asegurar una eficacia de protección entre 60 y 90% según si la duración es de 6 o 9 meses (10). En este contexto, y ante la ausencia de investigaciones locales previas, se planteó realizar este estudio para conocer principalmente el porcentaje de cumplimiento de TPI en los niños menores de 5 años contactos de pacientes con tuberculosis pulmonar confirmada por bacteriología en los establecimientos de salud de la ciudad de Quito y determinar los factores asociados.

## MATERIALES Y MÉTODOS

Se realizó una investigación operativa con diseño de cohorte. El momento de ingreso al estudio fue cuando iniciaron terapia preventiva con isoniacida y el momento final fue cuando terminaron el tratamiento o el momento en que dejaron de reclamar el medicamento en la institución de salud. Se tomó la información de las tarjetas de tratamiento diligenciadas entre enero de 2014 y diciembre de 2016.

La investigación se realizó en el Distrito Metropolitano de Quito, Ecuador, que corresponde a la Zona 9, capital del país, con una población de 2 645 509 habitantes en nueve distritos.

La administración del tratamiento antituberculosis en los pacientes ambulatorios se gestiona a través de los establecimientos de salud de primer nivel del Ministerio de Salud Pública (MSP), con 782 casos de tuberculosis que recibieron terapia en esta modalidad en el periodo 2014-2016 en 141 centros de salud de la ciudad de Quito.

La terapia preventiva con isoniacida se realiza a través de entregas semanales, quincenales, o ambas, en tomas no supervisadas, a los padres o tutores cuando la indicación está dada a los niños menores de 5 años contactos de pacientes con tuberculosis confirmada por bacteriología. De acuerdo a la normativa vigente en el país, se administra TPI 7 días a la semana durante nueve meses (36 semanas) (11).

### Población

La población del estudio estuvo compuesta por el total de niños menores de 5 años y los contactos de pacientes con tuberculosis pulmonar confirmada por bacteriología registrados en el Sistema de Información de la Estrategia de Prevención y Control de Tuberculosis de la Zona 9 (Quito) que iniciaron terapia preventiva con isoniacida. Se excluyó a los niños de quienes no se pudo obtener datos completos según la revisión realizada de las tarjetas de tratamiento y según las variables de interés (edad, sexo y distrito del caso índice y contacto, antecedente de TB, carga bacilar y condición de egreso del caso índice, año de inicio de TPI y cumplimiento del contacto), principalmente el número de dosis del medicamento entregadas por semana.

Para la evaluación de conocimientos se aplicó una encuesta a cuidadores definidos como personal de salud (entendido como funcionarios encargados de la Estrategia de Tuberculosis de los establecimientos de salud de primer nivel) y padres o tutores del contacto menor de 5 años que estén recibiendo TPI.

Las encuestas se aplicaron a los cuidadores identificados y de los cuales se pudo obtener información, de los niños que se encontraron en TPI durante el cuarto trimestre del año 2018. La participación en el estudio fue voluntaria.

### Variables de estudio y fuente de datos

En los menores de 5 años se tomaron variables demográficas y las relacionadas con el suministro de tratamiento, así como datos del caso índice que estuvieron disponibles en las tarjetas de control y administración de tratamiento y los informes mensuales.

Se utilizó también una encuesta de conocimientos elaborada *ad hoc* aplicada a los cuidadores de los niños menores de 5 años que recibieron TPI durante el año 2018.

La encuesta de conocimientos fue diseñada de acuerdo con la población, con preguntas enfocadas en la etiología, transmisión, tratamiento y el objetivo de la terapia preventiva con isoniazida. Estas preguntas se formularon con base en las definiciones del Manual de procedimientos del 2017 y la Guía de práctica clínica de tuberculosis del 2018 publicadas por el Ministerio de Salud Pública, el enfoque de las preguntas fue acorde con la población a la que serían aplicadas, personal de salud y tutores de los niños.

Las encuestas dirigidas al personal de salud fueron aplicadas por los responsables distritales de la Estrategia de Tuberculosis, incluyendo a la autora principal; y las encuestas dirigidas a los tutores fueron realizadas por el personal de salud de los establecimientos de salud, previo consentimiento informado.

La información recolectada se digitó en una base de datos y se codificaron las encuestas para conservar la confidencialidad de la información.

### Análisis de datos

Con base en los datos obtenidos se construyó la variable “cumplimiento de TPI”, mediante el uso de “número de semanas de entrega de TPI” y la convirtió a un porcentaje.

Se consideró como cumplimiento de TPI a quienes alcanzaron el 80% mínimo del tiempo indicado (8) que corresponde a nueve meses (36 semanas) según la normativa vigente, es decir al menos 28,8 semanas de terapia .

Las variables numéricas se describieron con mediana (rango intercuartil [RIC] Q1-Q3) y las variables cualitativas se describieron como frecuencia y porcentaje.

Para explorar la asociación del cumplimiento de TPI en relación con aspectos sociodemográficos y clínicos del caso índice y el contacto, se utilizó la prueba de chi cuadrado. Para evaluar la magnitud de la asociación, se utilizó la razón de prevalencia y se consideró como significativo un valor de *P* < 0,05.

Para el caso de la valoración de conocimientos de los cuidadores se utilizaron frecuencias absolutas para su descripción. Las variables cuantitativas como la edad se resumieron como mediana.

La investigación obtuvo la aprobación local del Comité de Bioética del Hospital Eugenio Espejo de la ciudad de Quito y del Comité de Ética de OPS, PAHOERC.

## RESULTADOS

**FIGURA 1:**
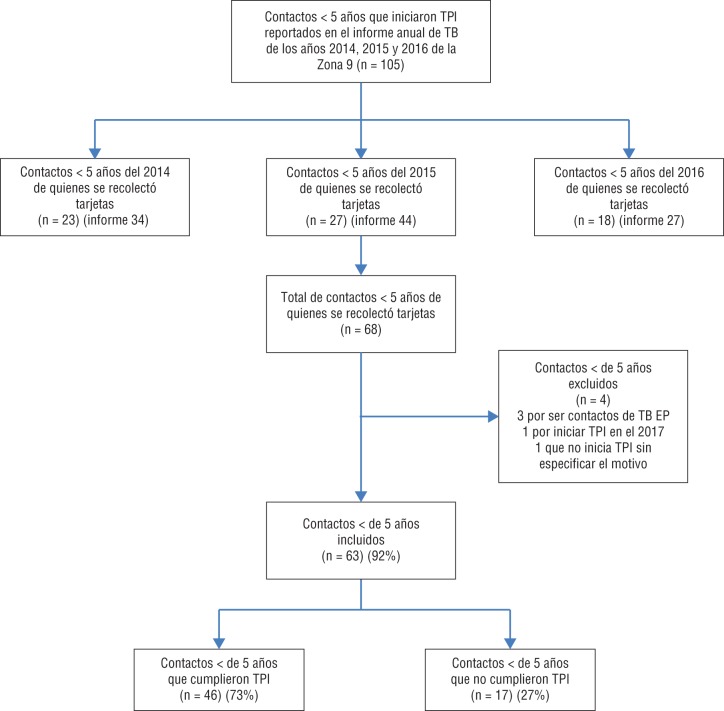
Proceso de recolección de información de los niños menores de 5 años contactos de pacientes con tuberculosis pulmonar confirmada por bacteriología que iniciaron TPI en la ciudad de Quito (2014-2016)

Se revisaron los informes anuales de los años 2014, 2015 y 2016, en los cuales se registraron 105 contactos menores de 5 años; sin embargo, se realizó el proceso de inclusión y exclusión como se describe en la figura 1.

Elaborado a partir de los resultados presentados.

TPI; terapia preventiva con isoniazida; TB, tuberculosis; EP, extrapulmonar.

Con respecto a los niños menores de 5 años que iniciaron TPI (n = 63), se identificó que los mismos correspondían a 44 casos índices, quienes presentaron un total de 215 contactos, 29,3% de los cuales eran menores de 5 años.

Del 2014 al 2016, de los 63 niños que iniciaron TPI, 73% la cumplieron. Se describe el cumplimiento como la toma de 80% o más de las dosis indicadas (9 meses = 36 semanas). Además, 88,9% de los niños completaron al menos 6 meses (24 semanas) de terapia.

La distribución de los contactos que recibieron TPI en los tres años fue de 20 en el 2014 (31,7%), 25 en el 2015 (39,7%) y 18 en el 2016 (28,6%). En cuanto a los niños menores de 5 años contactos que iniciaron TPI, se encontró que 52,4% (n = 33) son varones, con una mediana de edad de 20 meses (RIC: 8-36) en ambos sexos.

El sexo femenino fue predominante en los casos índices de los contactos en TPI, con 55,6% (n = 35), con una mediana de 35 años (RIC: 26-48) en ambos sexos. Se analizó, además, el antecedente de tuberculosis en los casos índices y se encontró que 6,3% (n = 4) de los pacientes refirió historia personal de algún tipo de tuberculosis; este porcentaje correspondía a recaídas.

En cuanto a la carga bacilar de los casos índices, el mayor porcentaje presentó una carga de 2+ (47,6%, n = 30). Se analizó, además, la condición de egreso de los pacientes como cumplimiento del tratamiento, para luego relacionarla con el cumplimiento o no de la terapia de los contactos; se identificó que 98,4% (n = 62) de los afectados terminó exitosamente el tratamiento; se consideró entre ellos a quienes egresaron como curados y como tratamiento completo.

La distribución de los casos en cada distrito fue variable, el distrito 7 el que presentó el número más alto de niños en TPI, con 28,6% (n = 18).

Al realizar el cruce de variables entre el cumplimiento o no de la TPI y las distintas características, se encontró que la carga bacilar del caso índice tiene una relación estadísticamente significativa en cuanto al cumplimiento de TPI de los contactos. Se observó que, a mayor carga bacilar, se tiene mayor porcentaje de cumplimiento: en el grupo de casos con TB pulmonar con baciloscopia (BK) 3+ al inicio de tratamiento, el 100% de cumplimiento de TPI de sus contactos.

Así también, la asociación de pertenecer a un distrito o no se relacionó con mayor cumplimiento de TPI (*P* < 0,001), como es el caso de los contactos pertenecientes a los distritos 7 y 9, en los cuales se presenta 100% del cumplimiento de la TPI. El año de inicio de TPI también presenta una relación estadísticamente significativa. Se observó que, durante el año 2016, 100% de los casos cumplen con el tiempo de TPI de al menos 80% de lo establecido (cuadro 1).

### Nivel de conocimiento de los cuidadores sobre TPI

#### Encuestas realizadas al personal de salud.

Se realizaron encuestas a 9 personas, funcionarios de los establecimientos de primer nivel de atención (centros de salud) que realizaron entregas de TPI a niños menores de 5 años, de los cuales 8 fueron mujeres (enfermeras) y 1 hombre (médico), con una mediana de edad de 36 años (23-52) y una mediana de 6 años de experiencia laboral (1-30).

En relación a las respuestas dados por los profesionales, se encontró que 2 de ellos determinaron al agente causal como un virus y 7 como bacteria; con respecto a la forma de transmisión todos reconocieron la forma aérea a través de la tos, aunque solo 7 a través de estornudos y al escupir. Todos identifican las siglas TPI como terapia preventiva con isoniacida, que debe administrarse durante 9 meses para evitar infección o enfermedad en los contactos menores de 5 años de pacientes con tuberculosis confirmada por bacteriología; sin embargo, solo 3 de los funcionarios identificaron al resto de grupos a quienes debe prescribirse la terapia (usuarios con VIH, personas que deben recibir terapia biológica y pacientes en diálisis y trasplante renal).

En cuanto a la forma de administración de la terapia, 8 personas indican que se realiza a través de los padres o tutores 7 días a la semana y 1 persona a través de observación directa por el personal de salud los 7 días. En cuanto a la dosis, 7 personas identificaron la dosis correcta de 10 mg/kg de peso. Además, se preguntó sobre los posibles efectos adversos de la isoniacida, 5 profesionales identificaron la totalidad de las posibles reacciones, 1 reconoció solo algunas y 2 no reconocieron reacciones.

#### Encuestas realizadas a los padres o tutores.

Se realizaron encuestas a 9 personas, tutores de los niños menores de 5 años en TPI, de los cuales 8 fueron mujeres y 1 hombre, con una mediana en edad de 32 años (26-42), 5 fueron madres de los niños, 3 padres y 1 tía; en cuanto al nivel de escolaridad, 2 sin instrucción, 2 con instrucción primaria, 3 con secundaria y 2 superior. Tres de los niños cuyos tutores fueron entrevistados se encontraban en el 1er trimestre, 2 en el segundo y 4 en el tercero.

En cuanto a las respuestas, se identificó que 7 personas reconocen al agente causal como una bacteria, 1 como un virus y 1 no sabe; en relación a la forma de transmisión, 8 personas responden que se realiza a través del aire al toser, 6 al estornudar, 4 al escupir y 2 por la saliva al besar; también 2 personas responden que la transmisión puede darse por compartir la misma vajilla. Los 9 cuidadores identifican a la isoniacida como el medicamento que reciben los niños, 8 con el fin de evitar que se contagien o enfermen y 1 no sabe; todos saben que los casos índice tienen tuberculosis pulmonar. Con respecto al tiempo de duración de la terapia, 7 personas reportaron que es de 9 meses, 1 de 6 meses y 1 no sabe; 8 responden que la forma de administración es de 7 días y 1 de 5 días a la semana por parte de los padres o tutores. Tres tutores mencionan todas las posibles reacciones adversas, 1 no sabe y 4 identifican algunas de ellas.

## DISCUSIÓN

Este es el primer estudio realizado en Ecuador sobre el cumplimiento de la TPI. Se realizó en Quito, la ciudad capital. Según la normativa vigente, la TPI tiene una duración de 9 meses con administración diaria, enmarcada en recomendaciones internacionales, como se menciona en la Guía de Manejo Programático de Tuberculosis Latente de la OMS 2018, en la cual se especifica esta alternativa en los países de baja carga de tuberculosis, dentro de los cuales se encuentra Ecuador (12).

Dentro de los hallazgos de la investigación se observa que se identificaron 215 contactos entre los 44 casos índices, lo que corresponde en promedio a 4,8 contactos por afectado de tuberculosis, dentro del rango esperado según la normativa (4 contactos por cada caso) (13).

**CUADRO 1.** Factores asociados al cumplimiento de TPI en niños menores de 5 años contactos de pacientes con tuberculosis pulmonar confirmada por bacteriología en la ciudad de Quito (2014-2016)

Variables	Cumplimiento de TPI						Valor de *P*^a^
	Sí (n = 46)		No (n = 17)		Total (n = 63)		
	n	%	n	%	n	%	
Sexo del contacto						
Femenino	23	76,7	7	23,3	30	47,6	0,534
Masculino	23	69,7	10	30,3	33	52,4	
Edad del contacto (meses) (Q1-Q3)	22	(5-36)	18	(12-36)	20	(8-36)	0,934
Sexo del caso índice							
Femenino	26	74,3	9	25,7	35	55,6	0,8	
Masculino	20	71,4	8	28,6	28	44,4
Edad del caso índice (años) (Q1-Q3)	34	(26-48)	35	(28-55)	35	(26-48)	0,261
Antecedentes de TB del caso índice							
Sin antecedentes	43	72,9	16	27,1	59	93,7	0,926	
Con antecedentes	3	75,0	1	25,0	4	6,3
Carga bacilar del caso índice							
1+	7	38,9	11	61,1	18	28,6	0,001					
2+	26	86,7	4	13,3	30	47,6
3+	9	100,0	0	0,0	9	14,3
Paucibacilar	0	0,0	1	100,0	1	1,6
Cultivo positivo	1	50,0	1	50,0	2	3,2
Sin datos	3	100,0	0	0,0	3	4,8
Condición de egreso del caso índice							
Curado	44	72,1	17	27,9	61	96,8	0,683		
Tratamiento completo	1	100,0	0	0,0	1	1,6
Fallecido	1	100,0	0	0,0	1	1,6
Distrito al que pertenece el caso							
2	2	28,6	5	71,4	7	11,1	< 0,001							
3	0	0,0	5	100,0	5	7,9
4	7	77,8	2	22,2	9	14,3
5	3	60,0	2	40,0	5	7,9
6	1	33,3	2	66,7	3	4,8
7	18	100,0	0	0,0	18	28,6
8	1	50,0	1	50,0	2	3,2
9	14	100,0	0	0,0	14	22,2
Año de inicio de TPI de los contactos							
2014	16	80,0	4	20,0	20	31,7	0,0001		
2015	12	48,0	13	52,0	25	39,7
2016	18	100,0	0	0,0	18	28,6

Elaborado a partir de los resultados presentados.

TPI, terapia preventiva con isoniacida; TB, tuberculosis; Q1-Q3, rango intercuartil.

^a^ Se utilizó la razón de prevalencia y se consideró como significativo un valor de *P* < 0,05.

El porcentaje de cumplimiento observado de 73% es similar al reportado en otros estudios, como en la investigación realizada en Brasil, en la cual 25,3% de 245 casos abandonó la terapia preventiva (75% de cumplimiento), y mayor comparado con los hallazgos de estudios efectuados en Colombia e Indonesia, donde el cumplimiento fue de 25,6% y 32%, respectivamente (6, 14, 15).

En Estados Unidos de América, se realizó un estudio para evaluar predictores de cumplimiento, con una tasa de 46,2%, menor que la mostrada en este estudio; para ello tomaron personas de todas las edades, sin encontrar diferencias por sexo (16). Otro estudio realizado en Noruega en 2016 incluyó el total de pacientes notificados con inicio de tratamiento (n = 726), de las cuales 62 eran menores de 5 años. En este estudio, el porcentaje de cumplimiento fue superior al presente, con 91% global. En cuanto a regímenes de tratamiento se encontró 89,5% de cumplimiento en quienes recibieron 3RH (3 meses de rifampicina e isoniacida) diario, 92,8% con 3RPH (3 meses de rifapentina e isoniacida) semanal y 80,9% con otros esquemas, dentro de los que se incluye isoniacida sola por 6 meses, similar al esquema que se indica en Ecuador. Además, se observó que la autoadministración disminuía la probabilidad de completar el tratamiento (17).

Los factores asociados a un mayor cumplimiento con significancia estadística (*P* < 0,05) fueron la carga bacilar del caso índice, el lugar de entrega y el año de inicio de TPI. Con respecto a la carga bacilar, se encontró que, a mayor carga del afectado por TB, mayor era el cumplimiento de la terapia preventiva de su contacto. No se hallaron estudios en relación con este hallazgo; sin embargo, podría estar en relación con una mayor percepción del riesgo de los contactos por parte de sus tutores, por la condición clínica y carga bacilar de los casos índices.

En relación con el lugar de entrega de la medicación, se encontró un mayor cumplimiento en dos distritos de salud (100%) y se verificó la carga de tuberculosis. El distrito 7 es el que cuenta con la segunda carga más alta de la ciudad con 11,7 casos por 100 000 habitantes. Además, aunque no fue una variable de estudio, se encontró que en este distrito la administración de la terapia en la mayoría de los casos era directamente observada al menos durante el tiempo de tomas del caso índice, factor que pudo haber influido para obtener un mayor porcentaje de cumplimiento. La forma de administración de la terapia se presenta también como un factor asociado a mejor adherencia en otras investigaciones, como la realizada en Perú por Chiang, en la que la mala adherencia a la TPI se determinó como una de las principales barreras para el manejo de la tuberculosis infantil y, dentro de sus causas, la administración no supervisada (7).

A pesar de que se encontró relación entre el año de inicio y el cumplimiento de TPI, no se identificaron diferencias en los componentes operativos en territorio que determinen esta condición.

Los conocimientos, actitudes y prácticas de las personas que intervienen en la administración de la terapia preventiva, que incluye a personal de salud y padres o tutores, se identifican como factores asociados al cumplimiento de las prescripciones en varios estudios. Estos factores influyen en la falta de percepción de la importancia de recibir la terapia por parte de los padres de los niños, en ocasiones por deficiencias en la explicación del personal de salud y creencias con relación a esta (7, 15).

Entre las limitaciones de este estudio se encuentra la inconsistencia de información entre los niños con TPI reportados en los informes trimestrales (n = 105) y la información obtenida de las tarjetas de tratamiento (n = 63), que corresponde a 60%; esto disminuyó, por ende, el número de registros a analizar.

En la ficha de tratamiento y en la historia clínica no se recoge información relacionada con los cuidadores, ni con las condiciones socioeconómicas del niño.

Otra limitación del estudio fue la relacionada con la aplicación del cuestionario a cuidadores y personal de salud, pues fueron pocos los niños en tratamiento en el último trimestre de 2018, y la tasa de respuesta del personal de salud que suministraba el tratamiento no fue la esperada.

### CONCLUSIONES

El mayor porcentaje de los niños menores de 5 años que iniciaron TPI cumplieron con al menos 80% de las dosis prescritas según la normativa. El cumplimiento estaba relacionado con condiciones del caso índice como la carga bacilar de diagnóstico, así como con el lugar de entrega de tomas y con el año de inicio de la terapia.

Con estos antecedentes, se sugiere realizar modificaciones en el sistema de información, como llevar un registro nominal estandarizado, que permitan un seguimiento adecuado del cumplimiento de la TPI en los niños, así como analizar la posibilidad de nuevas estrategias de tratamiento y administración, como tratamientos preventivos acortados y terapia directamente observada institucional o comunitaria, que garanticen la toma de la medicación; esto relacionado con la adopción y cumplimiento de protocolos y guías de práctica clínica.

Se deben plantear nuevas investigaciones operativas para ampliar el conocimiento sobre los factores asociados a la adherencia de TPI, la verificación de las tomas de la medicación y no solo de las entregas, el seguimiento prospectivo a los niños y la identificación del posible desarrollo de TB activa, así como sobre los conocimientos, actitudes y prácticas de los profesionales de salud, los afectados por tuberculosis, su entorno y la comunidad.

### Contribuciones de las autoras.

AC concibió el estudio original, recolectó y analizó los datos, interpretó los resultados y escribió el manuscrito. EA revisó el protocolo, interpretó los resultados y revisó el manuscrito. LL revisó el protocolo, procesó y analizó los datos, interpretó los resultados y revisó el manuscrito. Todas las autoras revisaron y aprobaron la versión final.

### Agradecimientos.

Las autoras agradecen al equipo del Ministerio de Salud Pública y OPS Ecuador por ser el nexo para realizar el curso SORT-IT que permitió la ejecución de esta investigación y en este contexto también a sus organizadores, tutores y compañeros por compartir tanto conocimiento y experiencias; finalmente al personal de los distritos y establecimientos de salud que participaron, pacientes y tutores de los niños.

### Declaración.

Las opiniones expresadas en este manuscrito son responsabilidad del autor y no reflejan necesariamente los criterios ni la política de la *RPSP/PAJPH* y/o de la OPS.

## References

[1] 1. World Health Organization. Global tuberculosis report 2018. Geneva: WHO; 2018.

[2] 2. Ministerio de Salud de Ecuador. Boletin Tuberculosis 2018. Disponible en: https://www.salud.gob.ec/wp-content/uploads/2019/03/informe_anual_TB_2018UV.pdf

[3] 3. World Health Organization. Guidance for national tuberculosis programmes on the management of tuberculosis in children. Geneva: WHO; 2015. 24999516

[4] 4. Cohn DL, O’Brien RJ, Geiter LJ, Gordin F, Hershfield E, Horsburgh C. Targeted tuberculin testing and treatment of latent tuberculosis infection. MMWR. 2000;49(6):1-54. 10881762

[5] 5. Szkwarko D, Hirsch-Moverman Y, Du Plessis L, Du Preez K, Carr C, Mandalakas AM. Child contact management in high tuberculosis burden countries: A mixed-methods systematic review. PLoS ONE. 2017;12(8). Disponible en: https://www.ncbi.nlm.nih.gov/pmc/articles/PMC5538653/10.1371/journal.pone.0182185 PMC5538653 28763500

[6] 6. Mendonça AMC, Kritski AL, Land MGP, Sant’Anna CC. Abandonment of treatment for latent tuberculosis infection and socioeconomic factors in children and adolescents: Rio De Janeiro, Brazil. PLoS ONE. 2016;11(5). Disponible en: https://www.ncbi.nlm.nih.gov/pmc/articles/PMC4858286/10.1371/journal.pone.0154843 PMC4858286 27149514

[7] 7. Chiang SS, Roche S, Contreras C, Del Castillo H, Canales P, Jimenez J, et al. Barriers to the treatment of childhood tuberculous infection and tuberculosis disease: a qualitative study. Int J Tuberc Lung Dis. 2017;21(2):154–160. 10.5588/ijtld.16.0624 28234078

[8] 8. Gomes VF, Wejse C, Oliveira I, Andersen A, Vieira FJ, Carlos LJ, et al. Adherence to isoniazid preventive therapy in children exposed to tuberculosis: a prospective study from Guinea-Bissau. Int J Tuberc Lung Dis. 2011;15(12):1637-43. 10.5588/ijtld.10.0558 22118171

[9] 9. Li J, Munsiff SS, Tarantino T, Dorsinville M. Adherence to treatment of latent tuberculosis infection in a clinical population in New York City. Int J Infect Dis. 2010;14(4):e292-7. 10.1016/j.ijid.2009.05.007 19656705

[10] 10. Horsburgh CR, Rubin EJ. Latent tuberculosis infection in the United States. N Engl J Med. 2011;364(15):1441-8. 10.1056/NEJMcp1005750 21488766

[11] 11. Ministerio de Salud Pública del Ecuador. GPC tuberculosis 2018. Disponible en: https://www.salud.gob.ec/wp-content/uploads/2018/03/GP_Tuberculosis-1.pdf

[12] 12. Wolrd Health Organization. Global Tuberculosis Programme. Latent tuberculosis infection: updated and consolidated guidelines for programmatic management. Geneva: WHO; 2018. Disponible en: http://www.ncbi.nlm.nih.gov/books/NBK531235/30277688

[13] 13. Ministerio de Salud Pública de Ecuador. Manual de Procedimientos para la Prevención y Control de la Tuberculosis. Ecuador; 2017. Disponible en: https://www.salud.gob.ec/wp-content/uploads/2017/07/MANUAL-DE-PROCEDIMIENTOS-DE-TB-FINAL.pdf

[14] 14. Castro RD. Programa de niños en contacto con adultos con tuberculosis en Popayán, Colombia.: Un estudio de cohorte. Rev Fac Cienc Salud Univ Cauca. 2014;16(2):24-30.

[15] 15. Rutherford ME, Ruslami R, Maharani W, Yulita I, Lovell S, Van Crevel R, et al. Adherence to isoniazid preventive therapy in Indonesian children: a quantitative and qualitative investigation. BMC Res Notes. 2012;5(1):7. 10.1186/1756-0500-5-7 PMC3287144 22221424

[16] 16. Stockbridge EL, Miller TL, Carlson EK, Ho C. Predictors of latent tuberculosis infection treatment completion in the US private sector: an analysis of administrative claims data. BMC Public Health. 2018;18. Disponible en: https://www.ncbi.nlm.nih.gov/pmc/articles/PMC5975486/10.1186/s12889-018-5578-3 PMC5975486 29843664

[17] 17. Schein YL, Madebo T, Andersen HE, Arnesen TM, Dyrhol-Riise AM, Tveiten H, et al. Treatment completion for latent tuberculosis infection in Norway: a prospective cohort study. BMC Infect Dis. 2018;18. Disponible en: https://www.ncbi.nlm.nih.gov/pmc/articles/PMC6245849/10.1186/s12879-018-3468-z PMC6245849 30453946

